# Adaptive evolution and early diversification of photonic nanomaterials in marine diatoms

**DOI:** 10.1038/s41598-024-82209-w

**Published:** 2025-02-21

**Authors:** Matt P. Ashworth, Daryl W. Lam, Martin Lopez-Garcia, Schonna R. Manning, Johannes W. Goessling

**Affiliations:** 1 UTEX Culture Collection of Algae, University of Texas at Austin, Austin, TX USA; 2 Department of Biological Sciences, University of Alabama, Tuscaloosa, AL USA; 3 Instituto de Òptica, Consejo Superior de Investigaciones Cientìficas (IO-CSIC), C/ Serrano 121, Madrid, Spain; 4 Institute of Environment, Department of Biological Sciences, Florida International University, Miami, FL USA; 5 Laboratory for Innovation and Sustainability of Marine Biological Resources (ECOMARE), Centre for Environmental and Marine Studies (CESAM), Department of Biology, University of Aveiro, Aveiro, Portugal

**Keywords:** Photonic crystal slab, Photonic stopband, Microalgae, Diatoms, Evolution of symmetry, Bioinspiration, Biophysics, Biotechnology, Evolution, Environmental sciences, Ocean sciences, Materials science, Optics and photonics

## Abstract

The discovery of natural slab photonic crystals in diatoms has sparked questions regarding their prevalence and functional role in nature. These advanced nanomaterials open so-called photonic stopbands in two dimensions, enabling precise light manipulation essential in technologies like quantum computing and photonics. However, the natural role of these advanced properties remains a mystery. In this study, we adopted a multidisciplinary approach, combining taxonomic, evolutionary, and photonic property analyses. Using scanning electron micrographs, we examined the girdle elements of the silicon dioxide shell from several hundred diatom species to explore the potential correlation between occurrence of slab photonic crystals and taxonomic relationship. Girdle slab photonic crystals emerged among the oldest diatom classes, with stopband properties covering the visible to mid-infrared spectral range. Square lattice formations emerged first, evolving from quasi-ordered templates, and later diverged into more efficiently packed hexagonal structures. This study shows how photonic nanomaterials developed from quasi order to order over evolutionary trajectories and provides a sustainable inventory of natural slab photonic crystals for application and deeper investigation. Understanding how diatoms utilize these structures can inspire innovative principles for smarter energy management systems as well serve as a model for the evolution of order and symmetry in cellular processes and organization.

**Supplementary Information:**

The online version contains supplementary material available at 10.1038/s41598-024-82209-w.

## Introduction

**Fig. 1:**
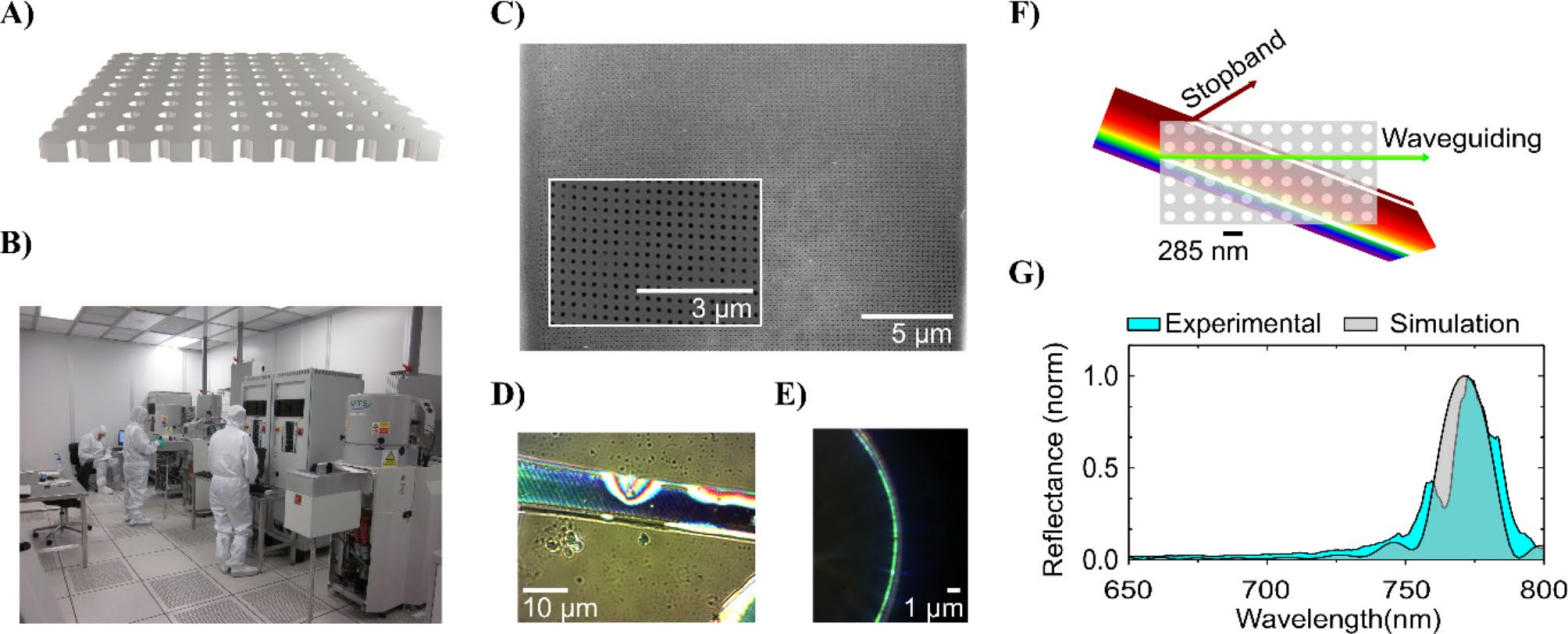
Identification of natural slab photonic crystals (sPhCs) post human invention. (**A**) Render of a common man-made sPhC. Conventional production of sPhCs begins with the computer-aided design of the photonic material. (**B**) Manufacturing involves cleanrooms, relying on trained personnel, advanced technology, and environmentally unfriendly methods. Typically, sPhCs are made from dielectric materials such as polymers, silicon, or silicon oxides. (**C**) The first natural sPhC was experimentally confirmed in the girdle band of the diatom *Coscinodiscus granii*. The inset zooms in on this highly ordered silicon dioxide nanomaterial with a pore-to-pore distance of ca. 285 nm. (**D**) Light diffraction and iridescence observed in *C. granii* girdle when flattened on a substrate under normal incidence illumination. (**E**) Waveguiding and light outcoupling in the green wavelength range, as expected for sPhCs with this period when immersed in water. (**F**) Schematic and simplified depiction of sPhCs spectral filtering properties: incident light is either reflected, transmitted or diffracted depending on the wavelength to period scale hence promoting certain spectral parts of electromagnetic radiation for propagation within the structure (waveguiding) while blocking others (stopband). These properties derive from interference processes attributable to the interaction of light with the nanostructure. (**G**) Reflectance measurements of the *C. granii* girdle band in water show a reflectance peak in the near-infrared (NIR), indicative of the photonic stopband characteristic of sPhCs. Experimental and simulated data are presented. Panel **G** is adapted from reference^8^ and reproduced under a common license agreement. Figure **B** shows some of the nanofabrication equipment available at the cleanroom facility of the International Iberian Nanotechnology Laboratory (INL) located in Braga, Portugal. Image reproduced with permission from the INL.

Bioinspiration has been a catalyst for cutting-edge technologies and the evolution of several scientific disciplines, including biomimetics and engineering^1^, evolutionary robotics^2^, artificial life^3^, synthetic biology^4^, and materials science^5^. The pursuit of more efficient, sustainable, and adaptable technologies rooted in biological principles has significantly impacted enabling technologies such as aviation, artificial intelligence, microfluidics, sensing, and photonics^6,7^. There are, however, instances where naturally occurring technology has only been recognized *post* human invention. An exemplary case is the discovery that diatoms naturally form advanced photonic structures found in their silicon dioxide shells that remarkably resemble the concept of slab photonic crystals (sPhCs) (Fig. 1).

Photonic crystals are periodic nanoscale structures for controlling the flow of light^9^. They have profound implications for some of the most advanced modern technologies, such as optical communications, high-performance lasers, efficient solar cells, ultra-sensitive sensors, quantum information processing, and more. Among the wide range of photonic crystal morphologies, the sPhC represents one of the most useful examples, by enabling light manipulation in up to three dimensions with relatively simple configurations^10^. The concepts facilitating the nanofabrication of sPhCs were initially developed in the late 1980s, opening new avenues for the precise engineering of light at nanoscale^11^. It was only after these advancements that similar structures were discovered in nature, specifically in the girdle band of the diatom frustule. This was first proposed through numerical simulations^12^ and later confirmed by experimental observations^8^. Diatoms (Bacillariophyta) – which diverged ~ 200 million years ago^13^ – predate human advancements in light manipulation for technology by hundreds of millions of years. However, at present, there are few hypotheses that explain the presence of these sophisticated photonic materials in diatoms^14,15^. Also, there is no clear understanding of how prevalent these structures are across the diversity of diatoms^16^ or if they could potentially exist as byproducts in only a handful of species without specific links to their biology and ecology.

**Fig. 2:**
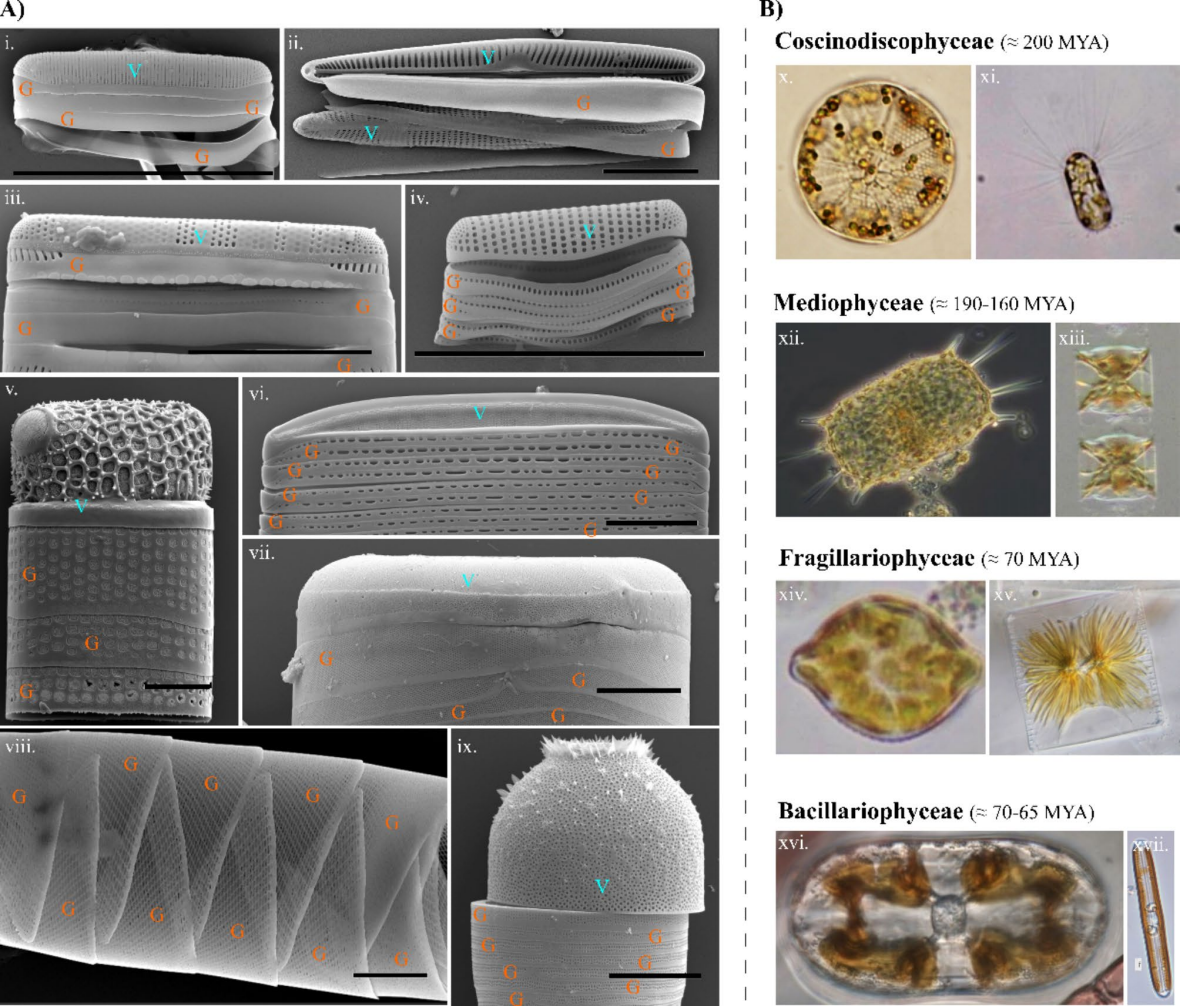
Exploring the ultrastructural diversity of diatom frustules from a taxonomic perspective. Diatoms are the most diverse group of phytoplankton, each possessing micrometre-sized frustules made of porous silicon dioxide. (**A**) Structural variety of diatom frustules, which are constituted of two components named valves (V) and girdles (G). (**B**) The extensive diversity in species, frustule shapes, and nano-perforations presents challenges in understanding the function and distribution of sPhCs in natural environments. To address this, we here adopted a taxonomic approach to identify the occurrence of natural sPhC morphologies according to taxonomic classifications. The four major clades of diatoms are presented, along with the evolutionary timeline indicating their first appearance. Species names: (i) = *Pteroncola inane*; (ii) = *Pseudogomphonema sp*.; (iii) = *Grammatophora* sp.; (iv) = *Placosira araneosa*; (v) = *Amphitetras antediluviana*; (vi) = *Nitzschia* sp.; (vii) = *Coscinodiscus* sp.; (viii) = *Rhizosolenia* cf. *imbricata*; (ix) = *Melosira* sp.; (x) = *Asteromphalus* sp.; xi. = *Corethron hystrix*; xii = *Trieres mobiliensis*; xiii = *Lithodesmium undulatum*; xiv = *Rhaphoneis amphiceros*; xv = *Striatella unipunctata*; xvi = *Amphora* sp.; xvii = *Navicula oblonga*. Scale bars in A) = 10 μm.

The diatoms are a photosynthetic microeukaryote group commonly found in both marine and freshwater environments. They are characterized by their distinctive silica shells named frustules. The frustule consists of two overlapping thecae, each composed of a valve and one or a series of overlapping girdle bands linking the valves to the other theca^17^. Beyond these broad characters, the frustules vary in overall and in fine morphology across the diversity of diatoms (Fig. 2). Frustules have therefore long served as the basis for the taxonomic classification of diatoms^17^. As the fine morphology of the frustule correlates to species identification in a group of organisms where species diversity is estimated to be in the tens of thousands or more^18^, there is significant variability in the distribution, density and morphology of features, such as the pores which perforate the frustule. Among various proposed applications of these natural nanomaterials^19,20^, the frustule, or at least parts of it, has also been considered as a photonic platform, where naturally occurring structural periodicity might drive the manipulation of light at nanometer scales^21^.

**Fig. 3:**
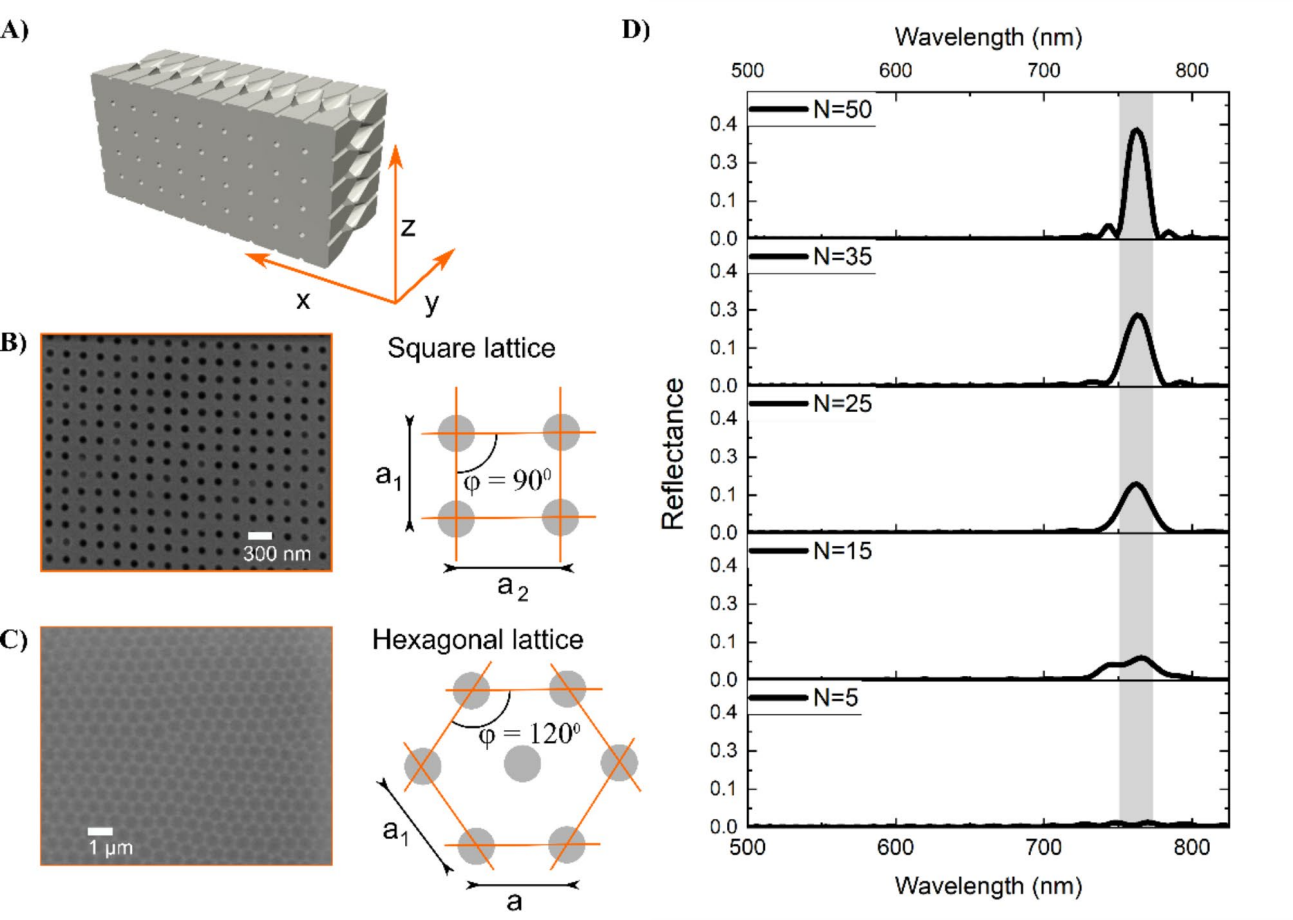
Identification of sPhC lattice symmetries in diatoms. (**A**) Illustration defining the directions along the expected pore symmetries in diatom girdles, which resemble sPhCs. This lateral pore arrangement has been previously identified in the species *Coscinodiscus granii*^8^ and was used to develop the model for characterizing sPhCs in this study. The internal pore system, as indicated in this computer-aided design, has a minor impact on the spectral position of the photonic stopband and was therefore not considered in the numerical analysis^8^. (**B**) Example of a square lattice sPhC identified in this dataset, as seen e.g. in the species *C. granii*. (**C**) Example of a hexagonal lattice sPhC, as observed e.g. in the species *Trieres mobiliensis*. (**D**) The number of pores in the z-direction significantly influences the reflectance intensity of the stopband. Our numerical analysis indicates that a minimum of 15 pores (N) in z-direction is necessary to observe stopband reflectance. Consequently, candidates with symmetrical pore symmetries having fewer than 15 pores were classified as quasi-crystals, as they lack the periodicity required to open a photonic band in z-direction.

Researchers have previously documented some optical characteristics of frustules cleared of their organic matter, as phenomena like iridescence become visible in dark field light microscopy^21^. Under certain lighting conditions, elements of the frustule can appear colored as a consequence of angular illumination despite the colorless nature of the silica core material^22,23^. Such photonic properties can manifest through various mechanisms, including light diffraction, scattering, or through refraction and reflection over porous features, particularly in the case of photonic crystal structures^24^. The foundation of these properties lies in the refractive index contrast between the photonic structure and the surrounding medium with a different refractive index. The number of repeated events over which such period exists builds the photonic response and controls its intensity^25^. While a single line of periodic pores (see e.g. Fig. 2A iii) can already possess photonic properties, sPhCs are characterized by highly periodic morphologies, commonly in 2 dimensions^26^. The photonic response of slab photonic crystals in the UV-VIS spectral range is due to light interference induced by features of a few hundred nanometres, similar to the wavelength of the visible light. The biosilica of the valve and girdle band slabs present periodically arranged structural features like pores which are filled with a different material, introducing refractive index contrast. The valve portion of the frustule has been most frequently documented as a photonic nanostructure^24,27,28^, and can, in some species ^[e.g. 12^, resemble the structural arrangement of complex 3D photonic nanomaterials^29^. However, the girdle bands of some taxa show more homogenous pore morphologies than the corresponding valves^8^. These girdle bands are likely to be the preferred unit for applications as sPhCs, as their overall perforation tends to be more uniform than that of valves and they lack additional ornamentation, such as siliceous cross walls, elevated pore fields or siliceous spines found on valves of some species. Girdle bands can exhibit intricate morphologies in some diatom taxa, featuring a complex porous lattice, which bears a striking resemblance to advanced photonic components such as the previously mentioned sPhCs^30^. Remarkably, it remains the sole nanostructure with a comparable architecture known in nature. Different girdle pore lattice types exist, including hexagonal type and square types^8^ (Fig. 3), which may allow for versatile light manipulation and control. The spectral response is primarily linked to the photonic crystal morphology including pore-to-pore distance (period) and pore diameter, the latter of which defines the volume into which the contrast medium can be inserted^31^. The number of pores is of equal importance, defining the intensity of the stopband reflectance (Fig. 3D). Despite such advanced photonic properties and potentially natural preservation, currently there is no profound hypothesis explaining how the girdle sPhC system could contribute to the physiological functions of live diatoms.

While there is a growing interest in exploring the photonic properties of the diatom frustule, whether for the aforementioned applications or to comprehend its role within the organism, these investigations have concentrated on a limited number of diatom genera. Yet, the frustule morphologies studied so far represent just a fraction of the diverse morphologies found among diatoms. Thus, hypotheses about the functional implications of the diatom frustule based on the presumed photonic properties of these tested strains, such as the attenuation of UV radiation^32,33^, or the manipulation of light to reduce the formation of oxygen radicals^18^ or to increase photosynthetic efficiency^34–36^, should really be limited to taxa with the specific tested morphologies, rather than broadly applied to all diatoms. Only a few studies have focused on girdle bands, finding that the tested species feature precisely defined uniform lattices of pores suggesting sPhC properties^12,37^. In particular, the lattice of pores in the girdle bands of *Coscinodiscus granii* creates a so-called photonic stopband; that is, a spectral band for which light propagation within the nanostructure is prevented, in this case for light in the near infrared spectral range, when immersed in water. On the other hand, light propagation in the green spectral range is promoted *via* waveguiding effects when the structure is immersed in water or other low refractive index contrast media^8^.

**Fig. 4:**
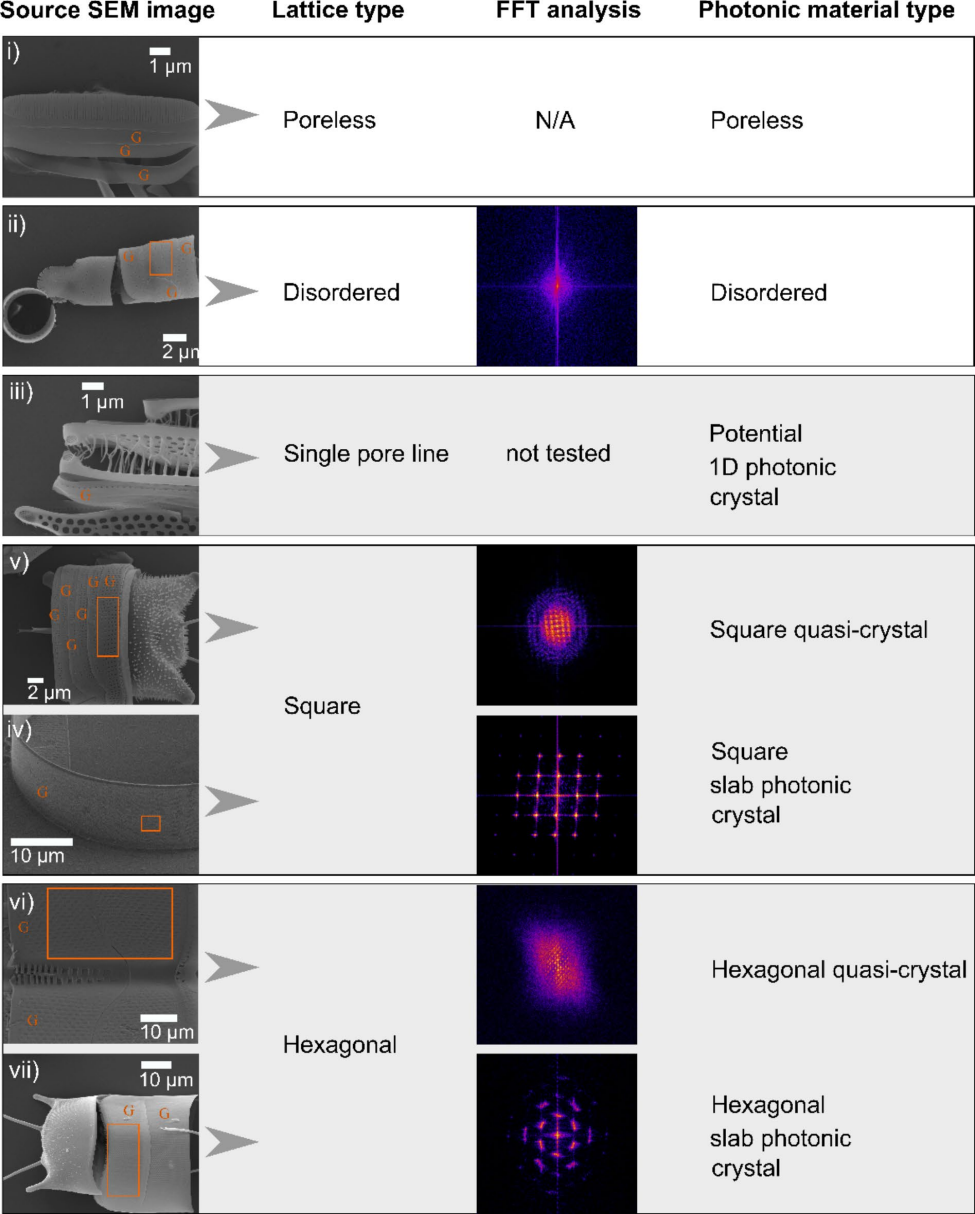
Lattice types identified in the girdle bands of diatoms. Girdle band structures are labelled as “G” in the source SEM images. Disordered structures lack symmetry, as indicated by the absence of distinct patterns in the Fast Fourier Transform (FFT) analysis. Single pore can be regarded to as potential 1D photonic crystals. Square and hexagonal lattice types may exhibit disordered features, evident as noise in the high-frequency domains of the FFT images. Species with such disordered lattices, here defined as quasi-sPhCs, contain fewer than 15 pores in the z-direction, such as those described in Fig. 3. Species names: (i) *Pteroncola sp.* UTKSA0078; (ii) *Orthoseira dendroteres* HK066; (iii) *Lambertocellus africana* SZCZP74; (iv) *Odontella sp.*; (v) *Coscinodiscus sp.*; (vi) *Triceratium spinosum* UTKSA0420; *Trieres mobiliensis*.

If these sPhCs provide some selective advantage, we would expect these properties to be common across diatoms, or at least trigger intense diversification in the clade where these properties evolved. Alternatively, if the selective advantage of photonic properties is limited, or restricted to a certain habitat, we would expect such lattices in only a few clades. To that end, here we have documented the ultrastructural characteristics of the girdle elements for hundreds of diatom species across the four broad taxonomic categories (Coscinodiscophyceae, Mediophyceae, Fragilariophyceae and Bacillariophyceae; Fig. 2). We developed a protocol to identify sPhC morphologies based on the periodicity and density of pores in frustule components (see Materials and Methods and Fig. 4). The evaluation of frustule morphology and photonic model parameters (specifically the stopband), alongside a molecular phylogeny of the examined diatoms, aimed to identify any patterns of correlation between sPhCs in the girdle bands and particular clades or ecologies.

## Results

**Fig. 5:**
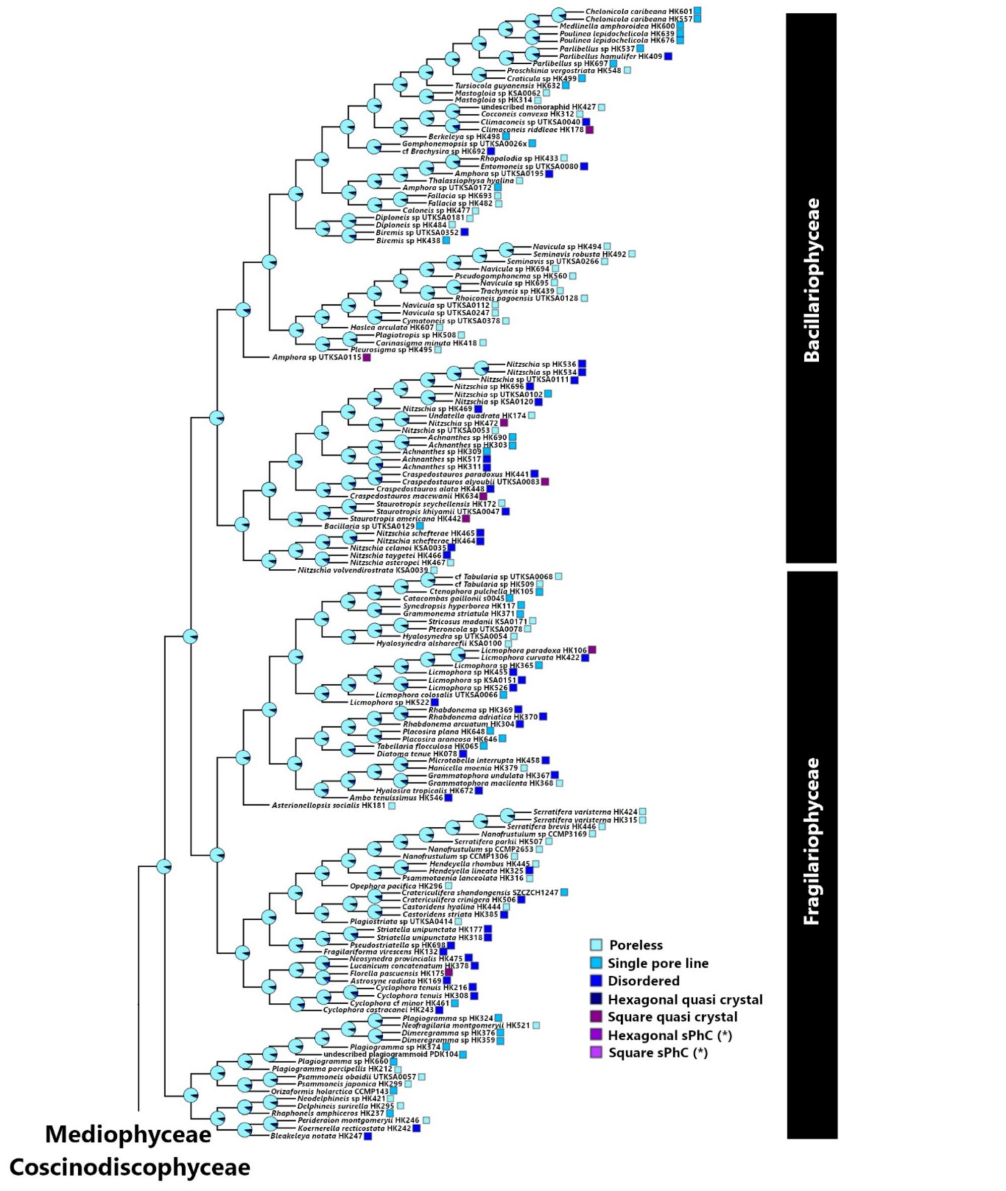

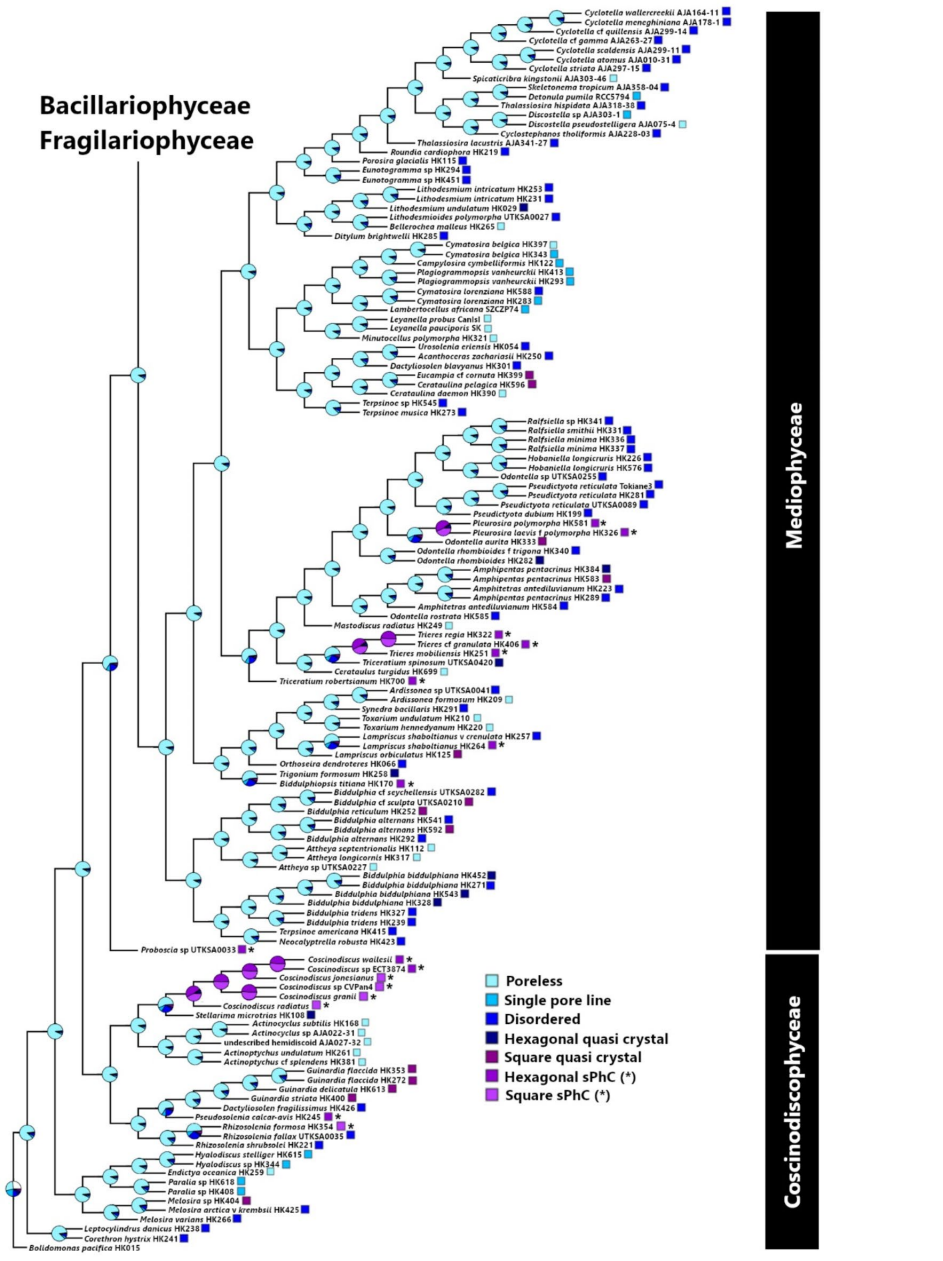
**A** Pore morphology distribution in girdle bands across Fragilariophyceae and Bacilariophyceae. Phylogenetic tree based on DNA sequence data from the diatoms evaluated for girdle band lattice morphologies. The likelihood of the distributions of specific lattice patterns in the girdle band poration are indicated at the nodes and branch tips. These lattice patterning states are labelled per the key provided at the right of the tree figure. The limits of the major taxonomic groupings are also illustrated at the right of the tree. **B** Pore morphology distribution in girdle bands across Coscinodiscophyceae and Mediophyceae. Square and hexagonal sPhC morphologies exist only within some clades within these two classes.

**Fig. 6:**
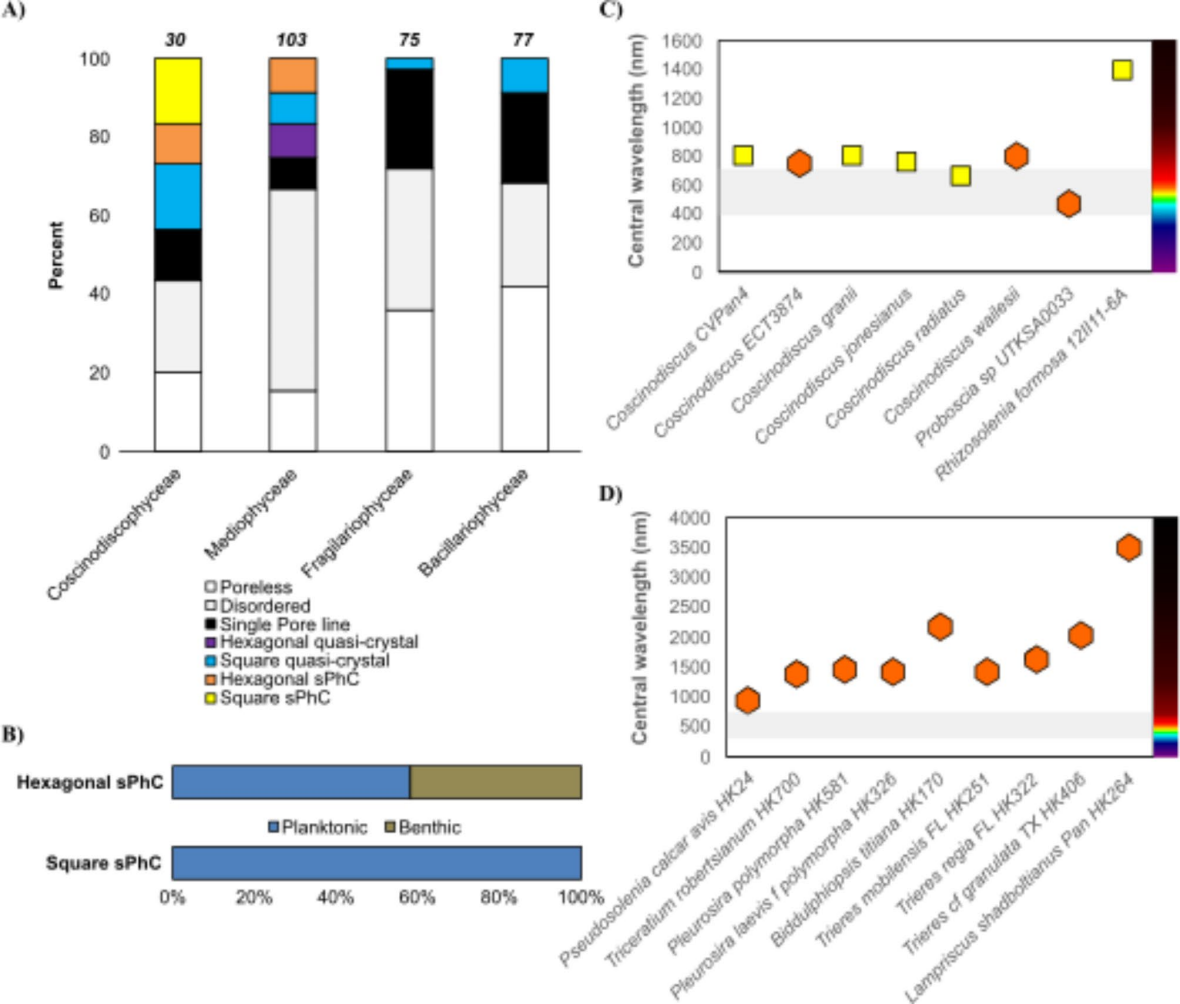
Spectral properties of photonic stopbands in diatom girdles identified as sPhCs. The lattice parameter, or the distance between adjacent pores, plays a crucial role in defining the spectral properties of the stopband, typically correlating with the spectral range of electromagnetic radiation. **A** Species exhibiting sPhC morphologies in their girdle bands are primarily found in the Coscinodiscophyceae and Mediophyceae clades, with Coscinodiscophyceae exhibiting exclusively square lattice symmetries. Disordered and poreless girdle bands become more prevalent in more derived clades. However, see Fig. S5 for a discussion of how this trend may have reversed, with sPhC properties shifting from girdles to valves in the Bacillariophyceae. The total number of species analyzed per clade is shown at the top of each bar. **B** All square lattice sPhCs identified in this study are associated with planktonic species, while hexagonal sPhCs are also found in benthic species. **C** Distribution of lattice parameters and central wavelengths of photonic stopbands in girdle bands of species from the Coscinodiscophyceae, oriented along the ΓX direction. **D** Distribution of lattice parameters and central wavelengths of photonic stopbands in girdle bands of Mediophyceae species, oriented along the ΓM direction. In **C** and **D**, the photosynthetically active radiation spectrum (λ = 400–700 nm) is shown as a grey horizontal bar. The corresponding spectrum is represented by a color scale on the right side.

The lattice arrangements and densities of pores are essential for the functioning of sPhCs^9^. For the girdle band to show sPhC properties, the silica frustule must present pores arranged in an ordered lattice arrangement in 2D. These lattice structures are unevenly distributed across the diversity of diatom girdle bands (Fig. 5A and B). Among the tested strains, taxa with potential sPhC girdle morphologies are exclusively found in the Coscinodiscophyceae and Mediophyceae classes (Figures S2 and S3, respectively), entirely absent in in both the Fragilariophyceae (“araphid pennates”) and Bacillariophyceae (“raphid pennates”) subclasses. These lattice structures appear in two broad categories: square and hexagonal lattices. While both types are present in the Coscinodiscophyceae, the square-lattice represents a larger proportion of the coscinodiscophytes, while the mediophytes only exhibit hexagonal-lattices (Fig. 6A).

It should be noted that while the Rhizosoleniaceae features taxa with lattice structures on their girdle elements, no sPhC candidates were identified among the strains. The genera *Proboscia* and *Pseudosolenia* Sundström appear to be the sole genera among the coscinodiscophycean clades which exclusively feature hexagonal latticesM. Among the mediophycean clades, two clades feature an abundance of lattice structures of both types. The aforementioned sPhC candidate *Trieres* is part of a larger clade of taxa in the Odontellaceae which contains additional genera bearing square (*Ralfsiella* [Ralfs] Sims, Williams & Ashworth) and hexagonal (*Pleurosira* [Meneghini] Trevisan) lattices. The genus *Biddulphia* Gray features both lattices, with *B. biddulphiana* (Smith) Boyer exhibiting a hexagonal-lattice and multiple square-lattices in the *B. alternans* (Bailey) Van Heurck/*B. reticulum* (Ehrenberg) Boyer clade. Hexagonal lattices are also found in *Trigonium* Cleve, *Biddulphiopsis* Stosch & Simonsen and *Lampriscus* Schmidt.

Regarding the cross-sectional ultrastructure of the girdle elements, all the sPhC candidates have loculate frustules, those types that possess chambers or compartments (Fig. S1/S2). Porose frustules dominate the phylogeny, while loculate frustules are distributed unevenly, concentrated highest in the Coscinodiscophyceae and only a few genera in the Mediophyceae (*Neocalyptrella* Hernández-Becerril & Meave, *Trieres*, *Trigonium*) and Bacillariophycidae (*Brachysira* Kützing, *Parlibellus* Cox). As with the lattice structures, however, not all taxa with loculate frustules are sPhC candidates, including those within the Rhizosoleniaceae. There is a distinctive shift towards unordered perforation, single row or poreless girdles at the Bacillariophyceae (Fig. 6A). Within the coscinodiscophycean clades, square-lattices are present at higher relative abundance in the Rhizosoleniaceae (*Rhizosolenia* and *Guinardia* H.Peragallo) and Coscinodiscus clades. Each of these clades do feature taxa with hexagonal-lattices as well (*Pseudosolenia calcar-avis* [Schultze] Sundström; *Coscinodiscus wailesii* Gran & Angst and *Coscinodiscus* sp. HK298, respectively).

On a finer scale, girdle elements with sPhC properties are found in a relatively small fraction of the sampled diatom diversity (Fig. 6A). In fact, only three clades were exclusively composed of taxa exhibiting sPhC properties in their girdle elements: the *Coscinodiscus* and *Proboscia* Sundström clades (Coscinodiscophyceae) and the *Trieres* Ashworth & Theriot + *Triceratium spinosum* Bailey clade (Mediophyceae). There were a few other sPhC candidates in the Coscinodiscophyceae (*Hyalodiscus stelliger* Bailey) and Mediophyceae (*Triceratium robertsianum* Greville and *Thalassiosira pseudonana* Hasle & Heimdal), but these were outliers among the other taxa in their respective clades. All identified square sPhCs were correlated to planktonic lifestyle, while hexagonal types could occur in both planktic and benthic species (Fig. 6B).

For taxa exhibiting sPhC-like lattice morphologies, analyzing the lattice parameter (period in z-direction) is crucial for inferring potential photonic properties and the spectral range using established models^8,31^. As depicted in Fig. 6C and D, species with square or quasi-square-lattices generally exhibit lattice parameters ranging from 100 to 300 nanometers, pertinent scales for eliciting photonic properties in the visible to near-infrared spectral range (450–1000 nm), according to models developed by the authors^31^. Figure 6C and D further illustrates the anticipated central wavelengths for the photonic stopband relative to the lattice parameter defined above. The stopband defines the spectral range in which light is impeded from propagating within the girdle band in this case along the ΓM and ΓX directions for hexagonal and square-lattices respectively (in z-direction ). Notably, the central wavelength falls within the visible to near-infrared range for most species with a square-lattice. Some species display larger values, resulting in significantly longer operating wavelengths for photonic properties. Interestingly, hexagonal-type lattices (Fig. 6D) exhibit a heterogeneous distribution of lattice parameters, ranging from lattice parameter ≈ 100 to ≈ 2000 nm with no apparent preferential range of values. This variability is reflected in a wide range of central operation wavelengths for the photonic properties, covering a slightly broader spectrum than square-lattices, extending up to 4000 nm wavelengths (mid-infrared).

## Discussion

The extensive variety of diatoms and their diverse frustule shapes have posed challenges to our understanding of photonic properties and if they may correlate with phylogeny and ecological niche occupation. In this study, we focused on exploring the presence of sPhC properties within the girdle bands, due to their more uniform morphology compared to the valves in many diatom species, examining both their diversity and taxonomic distribution. The sPhC properties emerge from highly geometrical periodic pore ornamentation, which we estimated using FFT image analysis over SEM micrographs. Our investigation revealed that sPhC properties are primarily confined to three clades of diatoms belonging to the relatively older evolutionary “centric” taxonomic groups: the Coscinodiscophyceae and Mediophyceae (Fig. S3 and S4). The more evolutionarily derived “pennate” clades, at least those tested in this study, did not exhibit sPhC morphologies within their girdle bands. Coscinodiscophyceae exhibited both lattice types of sPhCs, i.e. square and hexagonal structures, while Mediophyceae in this study showed solely hexagonal types.

**Fig. 7:**
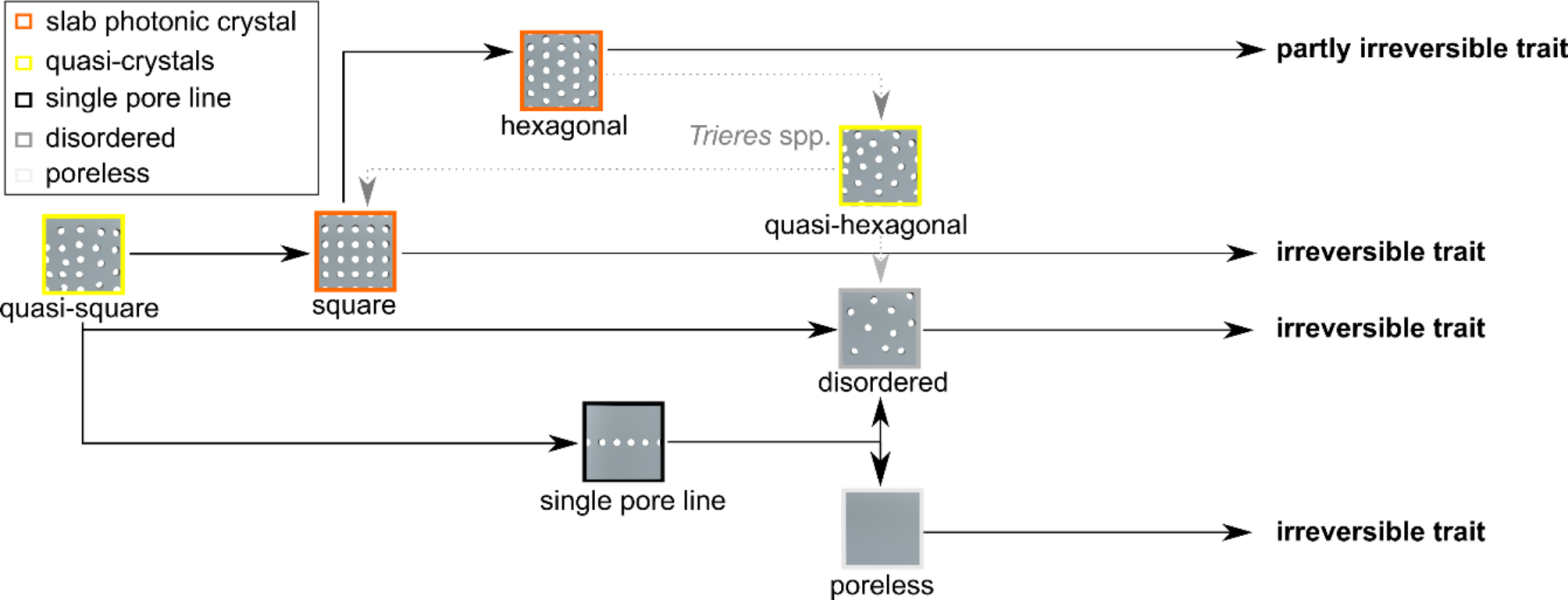
From quasi order to order, or disorder. Proposed trajectory of pore periodicity evolution in diatom girdle bands. The emergence of square lattice types appears to have occurred relatively early within the Coscinodiscophyceae, stemming from quasi-square lattice symmetries, as observed in extant species. Subsequently, hexagonal types arose from square lattice types. Both square and hexagonal types are reminiscent of sPhCs, while quasi-symmetries are quasi-photonic. Once established within a taxonomic lineage, square and hexagonal types appear to become irreversible traits. An exception exists within the *Trieres* family, where hexagonal types may partly exhibit quasi-hexagonal characteristics, potentially transitioning back to either square, or towards disordered types in some species. Unordered and pore less types similarly appear to persist as irreversible traits, once established. Boxes around the shapes indicate whether a structure is considered a sPhC (orange), quasi-photonic (yellow), single pore line (black), disordered (grey), or poreless (grey), following the definition developed in this study.

Our phylogenetic analysis provides important insight into the emergence of ordered nanomaterials, here exemplified by the sPhCs, which originate from quasi-ordered photonic structures positioned at the base of the phylogenetic tree (Fig. 5). In this context, we define quasi-order for structures that show symmetrical pattern, either square or hexagonal, but exhibit disorder such as seen in high-frequency domains in FFT analysis^38^ (as shown in Fig. 4). All girdles of species identified as quasi-crystals showed less than 15 pores in z-direction, here used as a proxy for the identification of sPhCs (see Fig. 3D). Due to their lack of periodicity in the z-direction, these quasi-crystals may not exhibit sPhC properties, which are defined by a 2D lattice constant. However, they can still function as 1D photonic crystals in x-direction. The influence of quasi-symmetry extends to potential asymmetry, resulting in the complete loss of pore features in girdles within the most derived evolutionary clades, specifically the Fragilariophyceae and Bacillariophyceae. Single pore lines, which may be regarded to as 1D photonic crystals, also increase in these clades (Fig. 6A). In summary, the trajectory of quasi-crystal order takes two divergent paths: it either evolved into (1) highly ordered sPhCs, or into (2) single pore lines or disordered lattice symmetries, which thereafter evolved into pore-less structures in the most recent groups of diatoms. Once a square sPhC morphology emerged at any juncture in the phylogenetic tree, however, subsequent lineages appear to retain this trait of high order, closing a return to quasi-order or asymmetry (Fig. 7). This observation implies that the evolution of square sPhC morphology imparts a selective advantage in a specific environmental context. This pattern is similar to one observed in correlated evolution studies looking at habitat and coloniality, which found that once a diatom clade switched from a planktonic to benthic habitat, the descendants rarely, if ever, returned to the plankton^39^. However, interestingly this is slightly different for hexagonal shapes, which emerge from square- or quasi-square ancestry, but can evolve back towards square-/quasi-square, or to disorder (Fig. 7, see *Trieres* spp.).

To investigate the potential biological functions of this observation further, we turn our attention to the distribution and anticipated photonic properties associated with square- and hexagonal-sPhC lattice types. Although both lattice types are present in the Coscinodiscophyceae, the more recent Mediophyceae is constrained toward hexagonal sPhC lattice type. This observation suggests a strong correlation between lattice type and phylogenetic lineage. Upon scrutinizing the predicted photonic properties of the square-lattice type, a particular pattern emerges. The anticipated spectral range of the photonic stopband predominantly aligns with the visible light spectrum in most cases of the square lattice type (Fig. 6C and D). This alignment could suggest a role in energy absorption in the visible spectrum of light, such as for photosynthesis or processes related to sensing visible light. In contrast, hexagonal-lattice types, characterized by larger lattice parameters, interact with the near and mid-infrared spectral range. Such properties may be associated with functions related to heat dissipation or the thermal regulation of cellular processes. While these hypotheses remain speculative at present, the intriguing correlation between lattice types, phylogeny, and distinct spectral ranges within the electromagnetic radiation spectrum prompts further exploration.

The absence of sPhC candidates within the girdle elements of the Bacillariophyceae can be readily attributed to the ultrastructure inherent in the girdle elements. Many of the girdle elements in bacillariophycean taxa only bear a single row of pores or lack perforation entirely (Fig. 6A). While a single line of pores can, under certain circumstances, create conditions for constructive and destructive interference of light due to periodic variations in the dielectric constant, it is classified as a 1D photonic crystal. This does not fulfill the criteria for 2D slab photonic crystals as examined in this study. The selective force, if any, driving this morphological shift from sPhC morphologies toward 1D photonic crystals or disorder, is unknown. It might be tempting to relate the raphe (the slit on the valve face that serves as the basis of motility for this subclass) to this change, hypothesizing that the evolution of motility in the diatom cell meant the diatom could reorient itself to alter light availability by a secondary derived trait and no longer required sPhC capabilities in the girdle elements. However, this shift towards single pore rows and unperforated girdle elements already appears to begin in the araphid pennate diatoms (Fragilariophyceae), which lack raphid motility.

With regards to ecology, many of the taxa with sPhC properties in the girdle elements are planktonic diatoms found in marine environments; however, some hexagonal types, restricted to some Mediophyceae, can also be found in benthic species. In fact, *Trieres* is one of the few planktonic genera in the Odontellaceae family (though *Triceratium robsertsianum*, a benthic taxon sister to the rest of the Odontellaceae, also exhibits sPhC properties). This dataset exhibits a notable bias towards marine benthic taxa, primarily during their greater availability during its construction. Additionally, it can be contended that there is also a strong bias in diatom diversity towards benthic taxa, given their presumed higher diversity and prevalence^40^. It can be anticipated that more of these sPhC structures exist in unstudied species. In our study, we also incorporated a less extensive dataset comprising selected valves of extant species. Interestingly, it seems that the transition from quasi-order to order follows a consistent logic and progression also in valves (Fig. S4). Particularly noteworthy is the observation that sPhC morphologies in valves are supposedly prevalent in more recent clades, specifically the Bacillariophyceae, suggesting a transfer of sPhC properties within this group from girdle bands to valves, possibly as a result of motility and new ecological niche occupation.

At this end, humans and diatoms stand out as the sole organisms capable of forming sPhCs. Artificial sPhCs find utility across a diverse array of technologies, including lasers^41^, quantum computing, quantum communication, quantum sensing^42^, phononic crystals for acoustic filtering and waveguides, phononic devices for vibration damping and acoustic cloaking, and thermal management applications^43^. Our research demonstrates the prevalence and diversity of sPhCs existing in nature, within the diatoms. However, whether or how diatoms use these structures, or if such morphologies evolved for a different reason, such as the provision of mechanical strength^44^, remain open questions. This study focussed on the girdle bands, finding that sPhCs in that specific structure are limited to the oldest clades, the Coscinodiscophyceae and Mediophyceae. Most of these natural sPhCs were found in the plankton, however, some mediophycean also showcased a benthic lifestyle, suggesting a transition of functionality while diverging into new habitats. We examined the stopband property, a parameter that has been previously studied experimentally in a single diatom species^8^. This parameter, however, is inevitably linked to the existence of guided modes. Future research should therefore also explore the potential implications of guided modes in this planar waveguide upon biological function within the organism. The correlation between lattice geometry and taxonomy spans across diverse clades and taxonomic groups of diatoms, persisting as an irreversible trait in most cases, with occasional exceptions, such as in *Trieres* spp. (Fig. 7). Our findings also indicate that highly ordered sPhC morphologies originate from quasi-ordered templates, with square lattice types emerging initially and persisting in younger clades of extant species. Notably, square sPhCs undergo diversification into hexagonal types, which boast a more efficient packing arrangement and, in the variety of species studied here, demonstrate interference with electromagnetic radiation in the mid- to near-infrared spectral range owing to larger pore periods. However, hexagonal types may undergo transitions into quasi-ordered or unordered structures in subsequent groups (i.e., *Trieres*), while square-lattice types seem to remain as an irreversible trait once established within a group.

Furthermore, our preliminary data suggest a comparable progression in valve morphologies, transitioning from disordered structure towards sPhCs topologies across taxonomic groups, with high symmetry seemingly shifting from girdle bands to valves in the more derived clades of the Bacillariophyceae (Fig. S4). We suggest including the analysis of valve nano-morphologies in future studies, not only to better understand the evolution of photonic structures in frustules but also to gain insights into the potentially more complex interactions between diatoms and electromagnetic radiation within these different components of the frustule. While our results open avenues for detailed investigations into the physiological implications of sPhCs on diatoms—supported by a comprehensive catalogue offering a range of structural lattice types and properties—it is currently limited to describing girdle band lattice constants, excluding valve morphologies, and encompassing 285 extant species (see Suppl. Table S1). However, as this list expands it may eventually encompass the total diversity of diatom frustule shapes, estimated to exceed 10,000 species^18^, each of which containing unique lattice symmetries, to choose from. Beyond contributing to our understanding of the distribution and evolution of periodicity in nature, exemplified by diatoms, this catalogue can also aid in the selection of dielectric nanomaterials with specific properties for ad hoc application. In combination of material properties and diversity, these materials hold promise as microelements for various applications, including bioinspired mechanic, microfluidic, photonic, phononic, sensing, as well as cutting-edge technologies such as nanomedicine, drug delivery, optoelectronics, and advanced materials science. A deeper understanding of the conditions under which sPhCs evolved, and their functional role in nature, may inspire novel mechanisms in light harvesting and energy management systems.

## Methods

### Morphological observations

The organic material was cleared from the frustules of existing diatom cultures at the University of Texas, Austin. Cells were treated in a 1:1:1 solution of cultured material:30% hydrogen peroxide:70% nitric acid for approximately 48 h, centrifuged at 4500 rpm for 20 min (TX-400 rotor, Sorvall ST16R Centrifuge, Thermo Scientific). The pellets were then rinsed in distilled water and repeatedly centrifuged until neutral pH was achieved. The neutral solution was then pipetted onto 12 mm diameter cover glasses and dried overnight. Cover glasses were subsequently mounted onto aluminum stubs and coated in 15 nm of iridium or platinum/palladium using a Cressington 208 Bench Top Sputter coater. Scanning electron microscopy (SEM) was conducted using a Zeiss SUPRA 40 VP scanning electron microscope (Carl Zeiss Microscopy, Thornwood, NH, USA).

### Identification of sPhC morphologies

The structural morphology of girdle bands was examined using size-calibrated SEM images. The SEM micrographs analyzed were from the four different taxonomic categories in an initial dataset containing Coscinodiscophyceae (*n* = 30), Mediophyceae (*n* = 103), Fragilariophyceae (*n* = 75), and Bacillariophyceae (*n* = 77). The corresponding categories were identified either with available DNA data or based on SEM morphological characterization.

The morphological analysis aimed to distinguish between square and hexagonal-lattice types, and to identify sPhC morphologies. The criteria included:

1. Exclusion of entries with girdle bands lacking pores or featuring a single line of pores radiating in the x direction, or unordered distribution of porous features. Note that a single line of pores can exhibit photonic properties, as seen in some 1D photonic crystals. However, these structures cannot be classified as slab photonic crystals, which require a 2D morphology^26^.
2. For entries with a periodic pore pattern in the x direction, determination of the number of pores in the z direction. For identification, crystal morphologies required a periodic pore pattern in at least two dimensions, with exclusion criteria of fewer than 15 pores in the z direction. This value based on numerical analysis, suggesting that at least 15 pores are needed for opening a photonic band (see Fig. 3D). These candidates were included in the identification of quasi-periodic morphologies. Specimens with more than 15 pores in each direction were classified as sPhCs.
3. The remaining SEM images from 2) underwent Fast Fourier Transform (FFT) image analysis using Fiji (ImageJ)^45^, a potent method converting spatial information into spatial frequency domains. FFT analysis provided information on the lattice structure and allowed us to exclude those species showing a lack of symmetry on their FFT image. Lattice morphologies (square or hexagonal) were differentiated by analyzing the normalized modules of the two lattice vectors (a_1_ and a_2_) and the angle formed between them (φ). A square-lattice is then defined by a_1_ = a_2_ and φ = 90^0^. Hexagonal-lattices were defined as a_1_ equals a_2_, and the angle φ equals 120 degrees. However, quantitative analysis was limited by the curvature often present in the girdle bands, or potential tilt of the natural structure in the SEM image. Despite this, the lattice type could still be clearly identified in the corresponding FFT images (see supplementary figures S2 and S3). In the case of a highly symmetric and orderly lattice of pores, multiple reciprocal lattice points exist within the FFT image. Each of these points corresponds to higher spatial frequencies, and their occurrence is more prominent as we move farther away from the central region of the image. The distance of each high intensity point to the center shows multiples of the periodicity of the structure in a given direction. In some cases, the lattice is very well-defined, and the pores are well ordered, which reflects on several reciprocal lattice points over a uniform background. In certain scenarios, the FFT analysis reveals distinct symmetry points set against a pronounced circular symmetry background, particularly at low spatial frequencies (Fig. 4; refer to quasi-crystals). This observation suggests the presence of areas with short-range order in the image, albeit alongside elements that exhibit disorder. Consequently, we have categorized these cases as square or hexagonal quasi-crystal (Fig. 3). We observed that all cases with high frequency domains in the FFT analysis, identified as quasi-crystals in this study, also exhibited fewer than 15 pores (See Table S1), reinforcing this numerically derived parameter as a reliable criterion for identifying sPhCs. It is important to note that in this context, we have labeled as quasi-crystal only those instances where a clear pattern was discernible in the FFT analysis. It is possible that additional quasi-ordered patterns may exist within this dataset due to variations in image quality.

All entries that successfully passed this scrutiny underwent analysis for pore period (in the x direction) and pore diameter. Additionally, we classified the types of pores, differentiating between regular pores, elongated pores, sieve pores, or other ornamental structures (Table S1).

### Numerical analysis of photonic crystal properties

The structural parameters of candidates demonstrating FFT symmetry, as outlined above, were used to compute the central reflectance of the photonic stopband. This computation incorporated refractive index approximations derived from models established for sPhCs in diatoms, as elaborated elsewhere^8^. It is important to acknowledge that the resulting central spectral reflectance values might not be entirely accurate, given the assumptions made during simulations, as observed in the case of species *Coscindodiscus granii*. Considering that the spectral range is predominantly influenced by the parameter pore period, the anticipated values are expected to align with these modelled data.

### Molecular phylogenetic analyses and ancestral state reconstruction

Among the strains used in the morphological analysis, 285 had corresponding DNA sequence data from the nuclear-encoded ribosomal small subunit (nrSSU), and the chloroplast-encoded *rbc*L and *psb*C genes generated for molecular phylogenetic studies. The DNA extraction, PCR amplification and Sanger sequencing protocols for these data are covered in Lobban et al.^46^. These loci were assembled into a concatenated dataset. All analyses for phylogenetic inference and ancestral state reconstruction were performed on the University of Alabama supercomputer cluster (UAHPC) (see Suppl. Table S2). Both Bayesian inference (BI) and maximum likelihood (ML) phylograms were inferred using ExaBayes^47^ and Iqtree^48^, respectively. For ancestral state reconstructions, data from comma separated value (csv) tables (Supplementary Table S2) were mapped onto the ML phylogram. For taxa with missing character state data in the csv file, the phylogram was pruned to match. Both discrete and continuous character sets were mapped via Phytools version 2.3.0 package^49^ as implemented through R version 4.4.1 ^50^. Model optimization for discrete character states were analyzed using the *fitMk* function in Phytools, that fits the *Mk* model to a phylogenetic tree and a discrete character state matrix under the equal rates (ER), symmetric rates (SYM), or all rates different (ARD) reversible models. Additionally, reversible ordered models of evolution were tested using *fitHMR* with the number of rate classes set to 1 (http://blog.phytools.org/2017/07/fitting-discrete-character-m-k-models.html). The best model was selected by a comparison of Akaike information criterion (AIC) scores of all the aforementioned models (Supplementary Table S3). The following best model was used for stochastic mapping: No_frustule <-> Poreless <-> Single Pore Line <-> Disordered <-> Hexagonal quasi-crystal <-> Square quasi-crystal <-> Heaxagonal sPhC <-> Square sPh. The *make.simmap* function (Phytools) was used in *parallel* (R) to stochastic map character states with a resulting total of 100,000 simulations for each csv file (http://blog.phytools.org/2017/11/running-makesimmap-in-parallel.html). For binary discrete character states, density maps were constructed using the *densityMap* function in Phytools.

## Electronic supplementary material

Below is the link to the electronic supplementary material.

Supplementary Material 1

Supplementary Material 2

Supplementary Material 3

## Data Availability

All data are available in the main text or the supplementary materials.
