# Author Correction: Adaptive evolution and early diversification of photonic nanomaterials in marine diatoms

**DOI:** 10.1038/s41598-025-92333-w

**Published:** 2025-03-04

**Authors:** Matt P. Ashworth, Daryl W. Lam, Martin Lopez-Garcia, Schonna R. Manning, Johannes W. Goessling

**Affiliations:** 1 UTEX Culture Collection of Algae, University of Texas at Austin, Austin, TX USA; 2 Department of Biological Sciences, University of Alabama, Tuscaloosa, AL USA; 3 Instituto de Òptica, Consejo Superior de Investigaciones Cientìficas (IO-CSIC), C/ Serrano 121, Madrid, Spain; 4 Institute of Environment, Department of Biological Sciences, Florida International University, Miami, FL USA; 5 Laboratory for Innovation and Sustainability of Marine Biological Resources (ECOMARE), Centre for Environmental and Marine Studies (CESAM), Department of Biology, University of Aveiro, Aveiro, Portugal

Correction to: *Scientific Reports* 10.1038/s41598-024-82209-w, published online 21 February 2025

The original version of this Article contained errors.

In the legend of Figure 7 the description of the colour labels was incorrect.

As a result,

“From quasi order to order, or disorder. Proposed trajectory of pore periodicity evolution in diatom girdle bands. The emergence of square lattice types appears to have occurred relatively early within the Coscinodiscophyceae, stemming from quasi-square lattice symmetries, as observed in extant species. Subsequently, hexagonal types arose from square lattice types. Both square and hexagonal types are reminiscent of sPhCs, while quasi-symmetries are quasi-photonic. Once established within a taxonomic lineage, square and hexagonal types appear to become irreversible traits. An exception exists within the *Trieres* family, where hexagonal types may partly exhibit quasi-hexagonal characteristics, potentially transitioning back to either square, or towards disordered types in some species. Unordered and pore less types similarly appear to persist as irreversible traits, once established. Boxes around the shapes indicate whether a structure is considered a sPhC (violet), quasi-photonic (dark green), or not photonic (ochre), following the definition developed in this study.”

now reads:

“From quasi order to order, or disorder. Proposed trajectory of pore periodicity evolution in diatom girdle bands. The emergence of square lattice types appears to have occurred relatively early within the Coscinodiscophyceae, stemming from quasi-square lattice symmetries, as observed in extant species. Subsequently, hexagonal types arose from square lattice types. Both square and hexagonal types are reminiscent of sPhCs, while quasi-symmetries are quasi-photonic. Once established within a taxonomic lineage, square and hexagonal types appear to become irreversible traits. An exception exists within the Trieres family, where hexagonal types may partly exhibit quasi-hexagonal characteristics, potentially transitioning back to either square, or towards disordered types in some species. Unordered and pore less types similarly appear to persist as irreversible traits, once established. Boxes around the shapes indicate whether a structure is considered a sPhC (orange), quasi-photonic (yellow), single pore line (black), disordered (grey), or poreless (grey), following the definition developed in this study.”

In addition, The Acknowledgements section in the original version of this Article was incomplete.

“The authors, specifically MPA, are grateful to the following researchers for their assistance in collecting material and cultures for electron microscopy and sequencing: E. Theriot, D. Williams, A. Witkowski, J. Witkowski, C. Lobban, R. Majewska, T. Frankovich, P. Sims, P. Kociolek, R. Jordan, S. Sato, A. Alverson, M. Sullivan, S. Bosak, J. S. M. Sabir. The authors, specifically, DWL thank Liam J. Revell for his assistance in mapping character trait data to the phylogeny analysis, and Michael R. McKain for his insightful discussions on the same topic. DWL is grateful to the University of Alabama Supercomputing Cluster (UAHPC) for computational time used for phylogenetic inference and character trait mapping.”

now reads:

“JWG is grateful for support from the Foundation of Science and Technology (FCT, Portugal) through a personal grant Concurso Estímulo ao Emprego Científico Individual grant (no. 2020.04217.CEECIND). JWG and MLG thank the FCT for their support through grant no. PTDCBTA-BTA20612021. JWG also thanks the FCT/MCTES for the financial support to CESAM (UIDB/50017/2020+UIDP/50017/2020+LA/P/0094/2020). MLG thanks the Research Council of Norway (NRC) for their support through the grant no. 342255. The authors, specifically MPA, are grateful to the following researchers for their assistance in collecting material and cultures for electron microscopy and sequencing: E. Theriot, D. Williams, A. Witkowski, J. Witkowski, C. Lobban, R. Majewska, T. Frankovich, P. Sims, P. Kociolek, R. Jordan, S. Sato, A. Alverson, M. Sullivan, S. Bosak, J. S. M. Sabir. The authors, specifically, DWL thank Liam J. Revell for his assistance in mapping character trait data to the phylogeny analysis, and Michael R. McKain for his insightful discussions on the same topic. DWL is grateful to the University of Alabama Supercomputing Cluster (UAHPC) for computational time used for phylogenetic inference and character trait mapping.”

The original Article has been corrected.

