# Supplementary material for: Adaptive evolution and early diversification of photonic nanomaterials in marine diatoms
Source: Sci Rep. 2025 Feb 21;15:6290. doi: 10.1038/s41598-024-82209-w (PMC11845468; doi:10.1038/s41598-024-82209-w)
Supplement: Supplementary file 1 — Supplementary Material 1 [file 41598_2024_82209_MOESM1_ESM.pdf]

**Revisions: Supplementary information**

# Adaptive evolution and early diversification of photonic nanomaterials in diatoms

Ashworth et al. 2024

## Supplemental figures

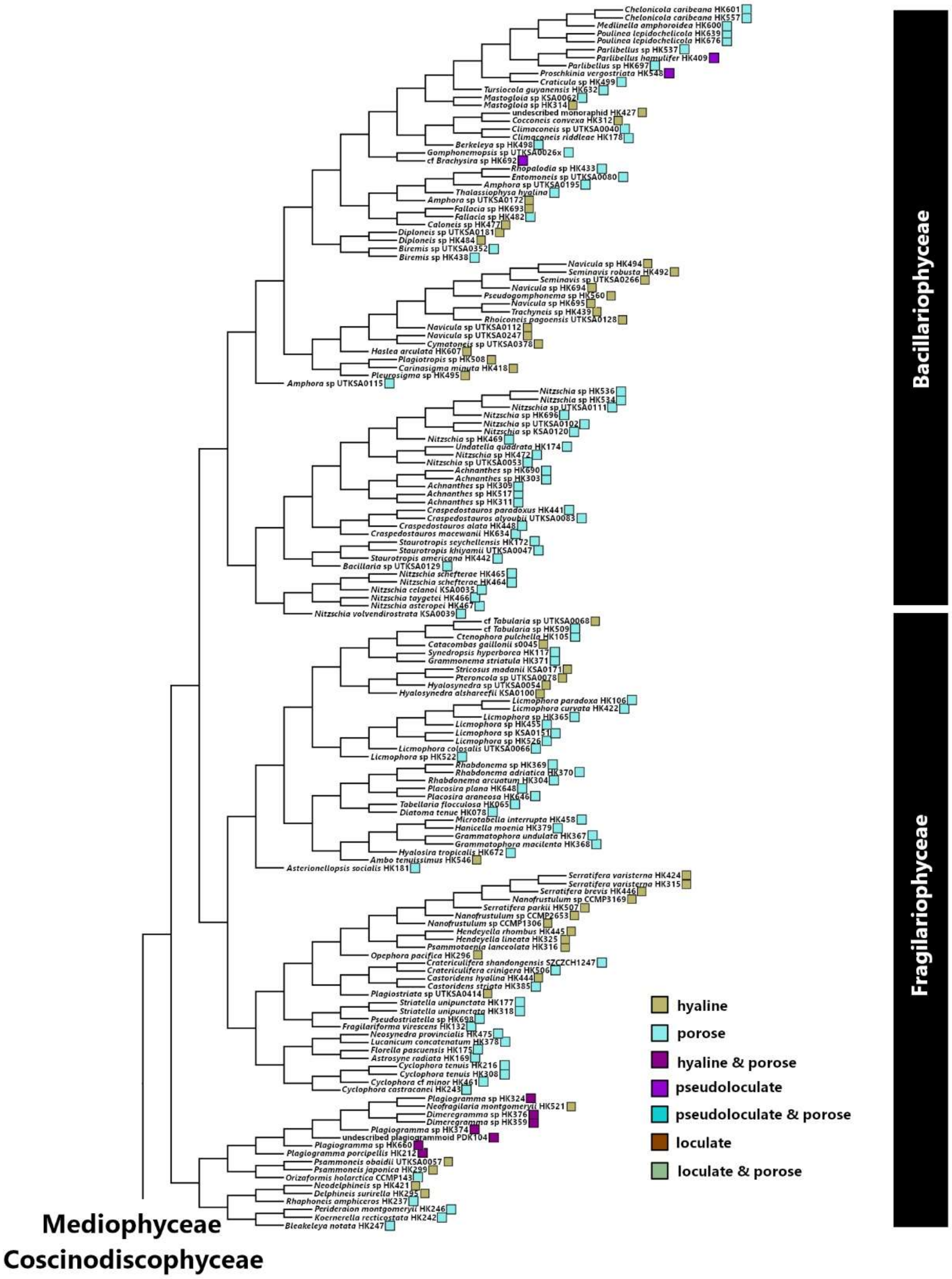


**Supplemental Fig. S1A. Phylogenetic tree based on DNA sequence data from the "pennate" diatoms evaluated for slab photonic crystal morphologies in girdle bands.** The tree was mapped with cross-dimensional morphology of the girdle bands (see legend). The cross-sectional morphology (see Supplementary Figure S2) of the girdle bands is indicated at the branch tips. Taxa marked with an asterisk (*) were identified as having slab photonic crystal properties in their girdle bands. The limits of the major taxonomic groupings are illustrated at the right of the tree.

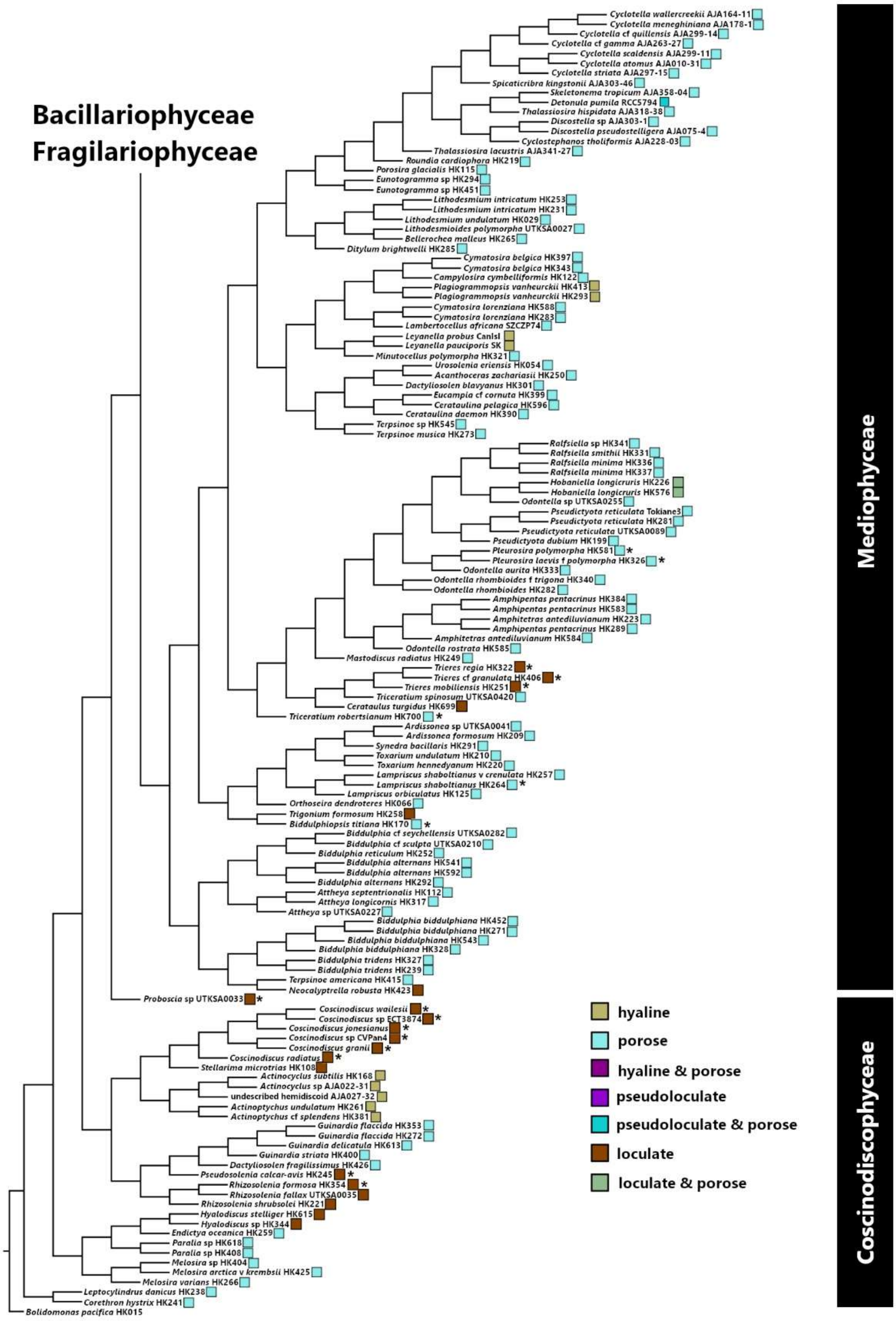


**Supplemental Fig. S1B. Phylogenetic tree based on DNA sequence data from the "centric" diatoms evaluated for slab photonic crystal morphologies in girdle bands.** The tree was mapped with cross-dimensional morphology of the girdle bands (see legend). The cross-sectional morphology (see Supplementary Figure S2) of the girdle bands is indicated at the branch tips. Taxa marked with an asterisk (*) were identified as having slab photonic crystal properties in their girdle bands. The limits of the major taxonomic groupings are illustrated at the right of the tree.

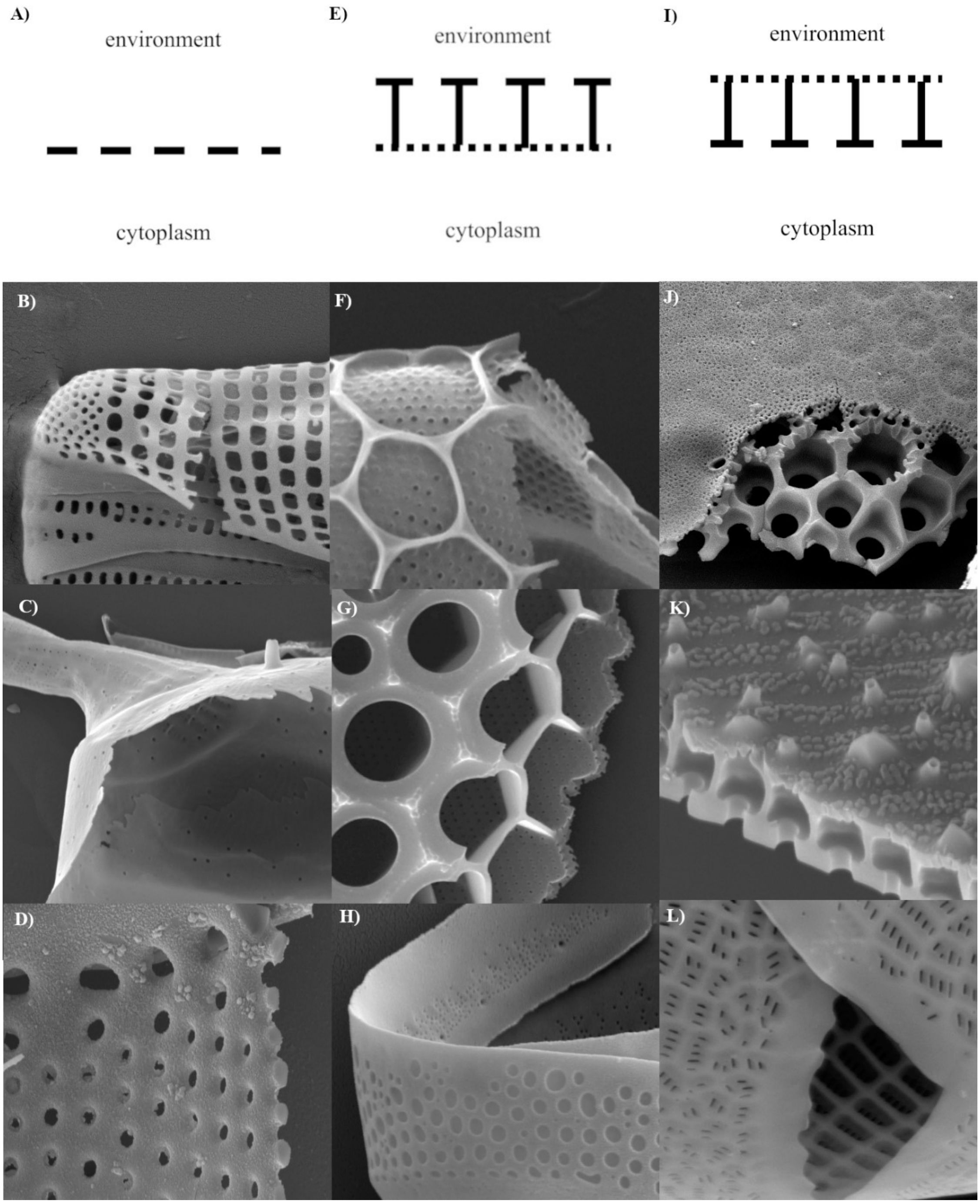


**Supplemental Figure S2: Types of cross-section morphology in diatom frustules.** The most common cross sectional morphologies of diatom frustules are porose (A-D), pseudoloculate (E-H) and loculate (I-L) valves and girdle bands. Whereas the porose frustule is composed of a single siliceous layer (A), the pseudoloculate (E) and loculate (I) frustules are essentially two-layered, chambered structures, where each chamber has a larger opening on the external (pseudoloculate) or internal (loculate) surface. B) *Placosira* sp. Lockport2 ribbonE1. C) *Chaetoceros* sp. UTKSA0020. D) *Biddulphia biddulphiana* HK328. F) *Stephanopyxis turris* HK213. G) *Endictya oceanica* HK259. H) *Thalassiosira* sp. AJA359-29. J) *Coscinodiscus* sp. HK263. K) *Cerataulus turgidus* HK699. L) *Rhizosolenia fallax* UTKSA0033.

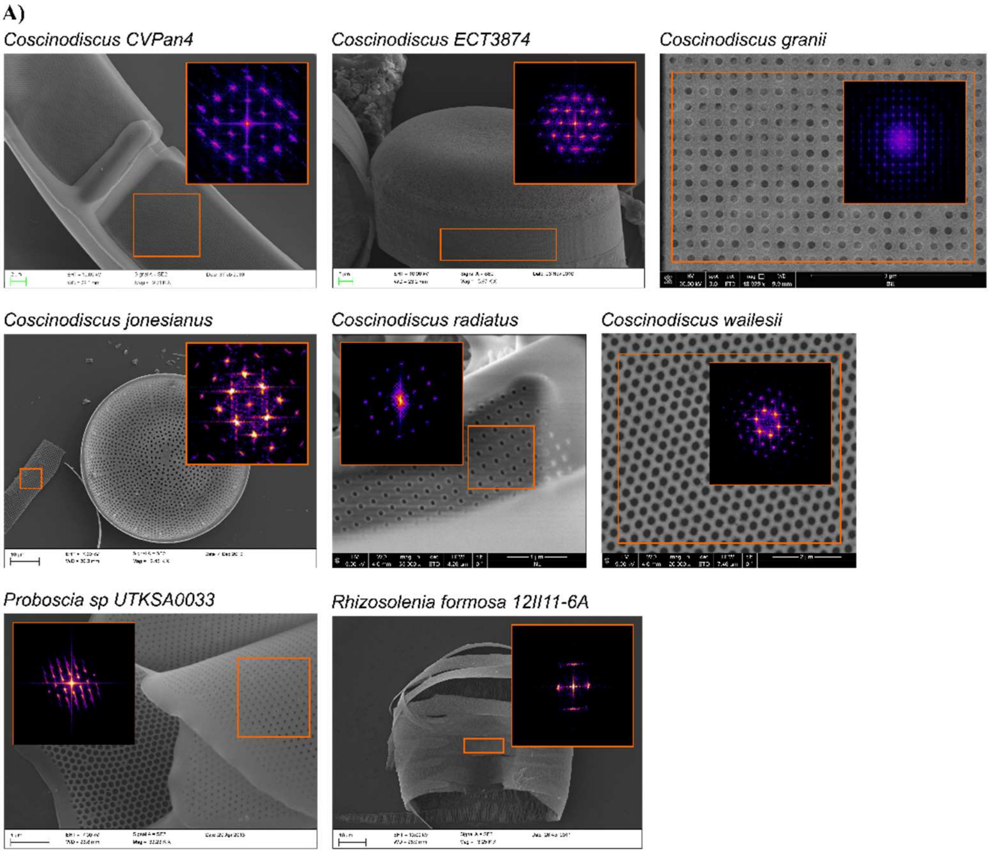


**Supplemental Fig. S3: Lattice symmetries of identified sPhCs in the Coscinodiscophyceae.** This group contains both lattice symmetries, square and hexagonal types. Note that 30 Coscinodiscophyeceae were analysis, of which 26.7 percent were identified as sPhCs. The area over which FFT analysis was performed is identified by an orange colored square.

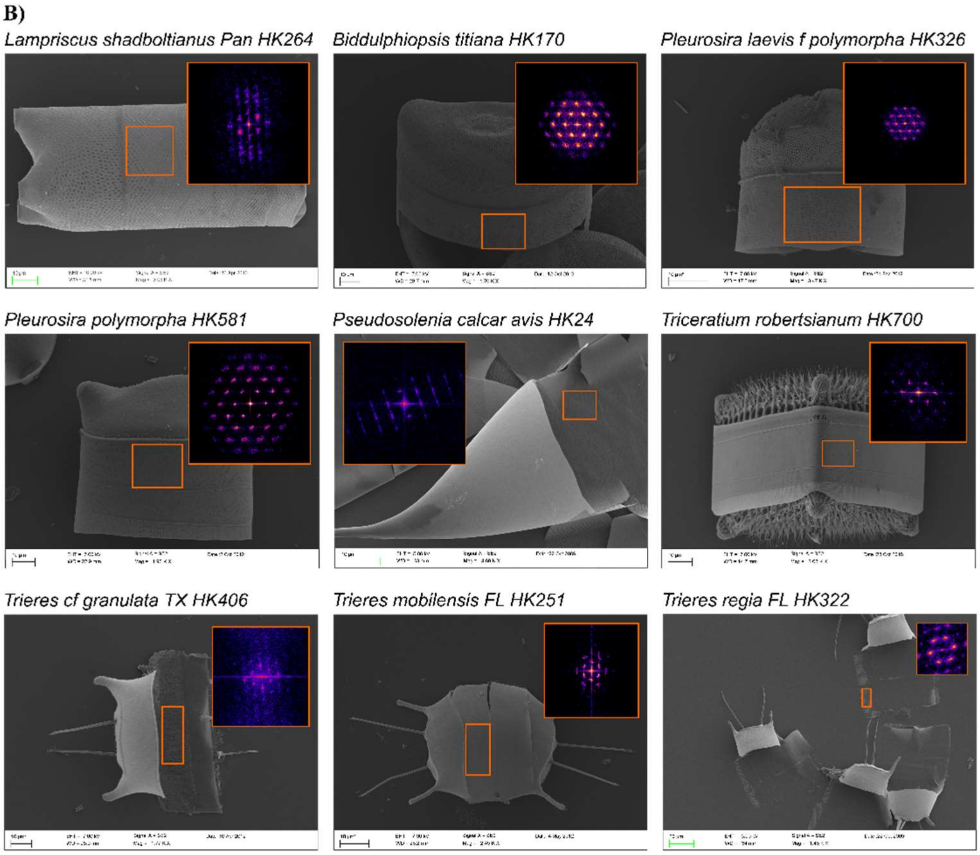


**Supplemental Fig. S4: Lattice symmetries of identified sPhCs in the Mediophyceae.** This group only contains hexagonal lattice types. Note that 103 Mediophyceae were analysis, of which 8.7 percent were identified as sPhCs. The area over which FFT analysis was performed is identified by an orange colored square.

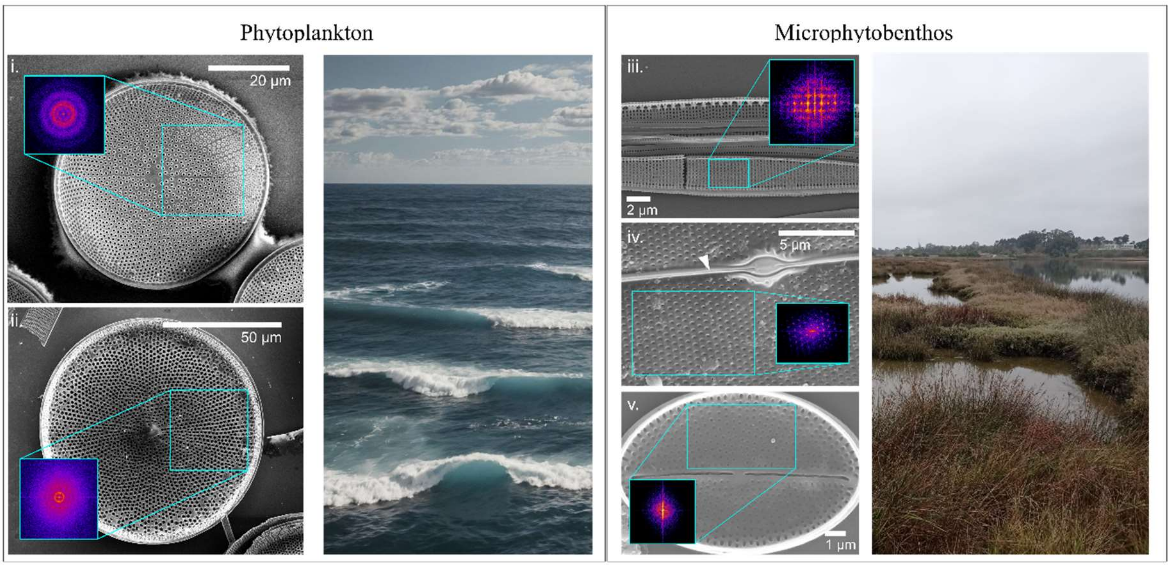


**Supplemental Fig. S5: Pore symmetries in valves across selected species of Coscinodiscophyceae and Bacillariophyceae.** While certain planktonic diatoms within the Coscinodiscophyceae exhibit sPhCs in their girdles, their valves may be considered disordered according to the definitions established in this study (as seen e.g. in i. *Coscinodiscus granii* and ii. *Coscinodiscus wailesii*). Conversely, raphe-bearing pennate diatoms inhabiting the microphytobenthos, characterized by motility via the raphe system (as indicated by the white arrow in iv.), within the Bacillariophyceae lack sPhC properties entirely in their girdles, yet some species exhibit sPhC properties in their valves (as shown in c. *Nitzschia* sp. and iii. *Pleurosigma* sp.). However, certain pennate raphids from this family do not display pore symmetry (*e.g.* v. *Cocconeis* sp.). This observation suggests a potential correlation between environmental stimuli and micro-niches, influencing the adaptation of sPhC properties, thereby facilitating niche differentiation.

## Supplemental tables and data

**Supplemental Table 1. Candidates with identified slab photonic crystal topologies in their girdle bands**.

**Supplemental Table 2. Akaike information criterion (AIC) scores for estimating the molecular phylogenetic trees**.
